# Distribution of Polyphenolic and Isoprenoid Compounds and Biological Activity Differences between in the Fruit Skin + Pulp, Seeds, and Leaves of New Biotypes of *Elaeagnus* *multiflora* Thunb

**DOI:** 10.3390/antiox10060849

**Published:** 2021-05-26

**Authors:** Sabina Lachowicz-Wiśniewska, Ireneusz Kapusta, Carla M. Stinco, Antonio J. Meléndez-Martínez, Anna Bieniek, Ireneusz Ochmian, Zygmunt Gil

**Affiliations:** 1 Department of Fermentation and Cereals Technology, Wrocław University of Environmental and Life Science, Chełmońskiego 37, 51-630 Wroclaw, Poland; zygmunt.gil@upwr.edu.pl; 2 Department of Food Technology and Human Nutrition, Faculty of Biology and Agriculture, Rzeszow University, Zelwerowicza 4, 35-601 Rzeszow, Poland; ikapustai@ur.edu.pl; 3 Food Colour and Quality Laboratory, Area of Nutrition and Food Science, Universidad de Sevilla, 41012 Seville, Spain; cstinco@us.es (C.M.S.); ajmelendez@us.es (A.J.M.-M.); 4 Department of Horticulture, University of Warmia and Mazury, Prawocheńskiego 21, 10-720 Olsztyn, Poland; anna.bieniek@uwm.edu.pl; 5 Department of Horticulture, West Pomeranian University of Technology in Szczecin, Słowackiego 17, 71-434 Szczecin, Poland; ireneusz.ochmian@zut.edu.pl

**Keywords:** carotenoids, cherry silverberry, seeds, polyphenolic compounds, antioxidative capacity, anti-diabetic activity, organic acids

## Abstract

The purpose of this study was to determine the distribution of polyphenolic and isoprenoid compounds and organic acids in the fruit skin + pulp, seeds, and leaves of six new biotypes of *Elaeagnus multiflora* Thunb., as well as their in vitro biological potency. The polyphenols and isoprenoids were determined with UPLC-PDA-MS/MS (ultra-performance liquid chromatography coupled to photodiode array detection and electrospray ionization tandem mass spectrometry) and RRLC-MS/MS (rapid resolution liquid chromatography/tandem mass spectrometry) methods, the organic acid with HPLC-RID (high-performance liquid chromatography coupled to a Refractive Index Detector), and the antioxidant capacity using ABTS and FRAP assays. Enzymatic activity was established as the ability to inhibit α-amylase, α-glucosidase, and pancreatic lipase. Owing to such an effective technique, 88 compounds were recorded, with 17 polyphenolic compounds and 3 isoprenoids identified for the first time in the seeds and leaves of cherry silverberry. In total, 55 compounds were identified in the leaves, 36 in the seeds, and 31 in the fruit skin + pulp. The predominant polyphenol was polymeric procyanidin (66–95% of total polyphenolics), whereas the predominant isoprenoids were chlorophyll b and (all-E)-lycopene. The results of our work noted that there are significant differences in the profiles of several secondary metabolites between the analyzed parts of the plant, and depending on the need, the compounds can be used to develop different innovative food or cosmetic products.

## 1. Introduction

*Elaeagnus multiflora* Thunb., appearing in the literature as goumi, cherry silverberry, or cherry elaegnus, belonging to the family Oleaster (*Eleagnaceae*), like the popular *Hippophaё rhamnoides* L. [1], is a thorny, broadleaved shrub reaching 3 m in height. It produces yellow or orange-red elliptical 1-cm-long fruits, which are juicy and have an astringent taste resembling that of red currant. The fruits are suitable for direct consumption. In addition to lipids, organic acids, and macro- and microelements, they contain significant amounts of lycopene. This carotenoid is responsible for their red color and has been extensively studied as one of the major dietary carotenoids for humans [2]. Lee et al. [3] demonstrated that in addition to chlorophylls, the leaves of cherry silverberry have high contents of sugars, fatty acids, and phytosterols. However, the polyphenolic profile of cherry silverberry fruits has not yet been thoroughly recognized. Only 14 compounds belonging to phenolic acids and flavan-3-ols have been identified so far [3]. Polyphenols are important compounds due to their beneficial effects, including anticarcinogenic, anti-inflammatory, antioxidant, antitumor, and antidiabetic, as well as anti-atherosclerotic, activities [4,5]. In addition, they are the focus of interest in the food industry due to their high nutritive value [3].

*E. multiflora* Thunb. is known in Chinese medicine as a therapeutic plant [6]. Its fruits, in both fresh and processed forms, are used to treat ailments such as cough, diarrhea, pruritus, gastrointestinal tract disorders, and even cancer [4]. *E. multiflora* Thunb. fruits are also used to make juices, jams, compotes, or jellies [1], while in Asia and eastern Europe, they are used to produce herbal teas, wines, desserts, soups, ice cream glazes, and even candies and puddings [7].

The cherry silverberry shrub is a native plant of China, Japan, and Korea. Not long ago, this species also appeared in Russia, Ukraine, and the United States, while since the 1990s, it has been investigated at the Department of Horticulture of the University of Warmia and Mazury in Olsztyn (Poland). Studies conducted at the department have mainly focused on the selection and development of novel cultivars perfectly coping with the moderate climate in Poland during cultivation [1]. The environmental factors are inseparable elements that affect the contents of biologically active components. The accumulation of polyphenolic, terpenoid, or polysaccharide compounds in a plant can be effective only upon sufficient access of light, phosphorus content in the soil, and—last but not least—soil organic matter [8]. As demonstrated by Lachowicz et al. [5], contents of the basic physicochemical compounds were significantly affected by the novel cultivars analyzed. In addition, these cultivars can be excellent components of health-promoting and other beneficial products intended for human consumption (functional foods, nutraceuticals, supplements, nutricosmetics, etc.) [9]. Recently, foods rich in polyphenols have attracted great interest due to their potential benefits for human health. Being present not only in fruits and vegetables but also in seeds, olive oil, and beverages such as coffee and tea, raw materials are fine elements of healthy, nutritional patterns. The latest evidence suggests that a high intake of polyphenols with a diet may be negatively correlated with the general mortality rate associated with diseases of the circulatory system, certain types of cancer, cardiovascular diseases, adverse anthropometric changes, and mood disorders [10]. All these traits make polyphenols potential elements in designing functional food and in developing prophylactic strategies for lifestyle diseases. The positive impact of polyphenolic compounds on health is due to several factors, the major ones of which include their content in diet and their bioavailability, which may differ significantly. The content of polyphenols in food may be influenced by genetic, environmental, and technological factors; some of these factors can be controlled to optimize the polyphenol content in food. One of the means to increase the polyphenol content in food may involve the appropriate choice of fruit and vegetable cultivars, which differ significantly in terms of their polyphenol content.

Biologically active compounds found in the cultivars (Jahidka and Sweet Scarlet) of cherry silverberry fruits have been identified [7]. However, little is known about the bioactive potential of the fractions of six novel biotypes of *E. multiflora* Thunb. cultivated in Poland. These studies tried to determine the versatile use of cherry silverberry in various areas of human life. Therefore, the objective of the present study was to characterize and determine the location of polyphenolic, carotenoid, chlorophyll, and tocopherol compounds (with ultra-performance liquid chromatography coupled to photodiode array detection and electrospray ionization tandem mass spectrometry and rapid resolution liquid chromatography/tandem mass spectrometry methods) and organic acids (with the high-performance liquid chromatography coupled to a Refractive Index Detector method) and their biological activity in the fruit skin + pulp, seeds, and leaves of six new biotypes of *E. multiflora* Thunb. cultivated in Poland. Results from this study (1) can be used in plant genetics to achieve new suitable biotypes, (2) determine the location of individual compounds in tested materials and, depending on the need, develop novel products for human consumption (fortified foods, functional foods, (nutri)cosmetics, etc.), and (3) could help in the management of by-products.

## 2. Materials and Methods

### 2.1. Plant Materials

Cherry silverberry plants were collected from the University of Warmia and Mazury in Olsztyn, Poland (53°50′ N, 20°31′ E), in June, 2019. The study used three vegetatively propagated biotypes (9-19-1996 (si1), 9-24-1996 (si2), and 9-34-1996 (si3)) obtained from the Institute for Fruit Growing in Samokhvalovitchy from the E-2 breeding farm and two biotypes (01-1999 (si0): 9-44-1996 (si4) and 9-84-1996 (si5)) obtained from the seeds of the 01-1999 (si0) biotype in 2005. The comparison of the biotypes also included the evaluation of the 01-1999 (si0) biotype obtained from seeds originating from a shrub cultivated in Olsztyn in 1999. All the plants were adults. 

All plants were planted with 4  ×  2 m^2^ spacing in Albic Luvisolx (Arenic) soil, which was deeply flattened and produced from clays of pH 6.8 in KCl [5]. The shrubs started to fructify in the third year after planting. This region of Poland is one of the coldest, and despite this, the studied biotypes adapted to this climate; therefore, it can be suggested that they would be suitable for cultivation throughout Europe. In the course of the tests, 3 replications (for each 25 randomly chosen leaves and about 2.5 kg of fruit) from 3 shrubs of each biotype were determined. The powders were stored in a refrigerator (−26 °C) until extract preparation. The raw material was directly frozen in liquid nitrogen and freeze-dried (24 h; Christ Alpha 1-4 LSC; Osterode am Harz, Germany). The homogeneous dry material was obtained by crushing the dried tissues using a closed laboratory mill (IKA A.11; Darmstadt, Germany). The powders were kept in a refrigerator (−80 °C) until extract preparation [7].

### 2.2. Identification and Quantification of Polyphenols

The extraction and measurement of polyphenolic compounds by the UPLC-PDA-MS/MS technique were performed according to the method by Kapusta et al. [11]. Separation was carried out using a BEH C18 column (100 mm × 2.1 mm i.d., 1.7 µm; Waters) kept at 50 °C. For polyphenolic compounds, a lower concentration of formic acid was used (0.1% *v/v*). The gradient program was set as follows: 0 min, 5% B; from 0 to 8 min linear to 100% B; and from 8 to 9.5 min for washing and back to initial conditions. The injection volume of the samples was 5 µL (partial loop with needle overfill), and the flow rate was 0.35 mL/min. The following parameters were used for TQD: capillary voltage, 3.5 kV; con voltage, 30 V in positive and negative modes; source temperature, 250 °C; desolvation temperature, 350 °C; con gas flow, 100 L/h; and desolvation gas flow, 800 L/h. Argon was used as a collision gas at a flow rate of 0.3 mL/min. 

Polyphenolic detection and identification were based on specific PDA spectra, mass-to-charge ratio, and fragment ions obtained after collision-induced dissociation (CID). Quantitative analysis was based on specific MS transitions in multiple reaction monitoring (MRM) mode. The MRM transitions, cone voltage, and collision energy of each individual polyphenolic compound were set manually with a dwell time of at least 25 ms. All measurements were noted three times and expressed as mg/100 g dry matter (d.w.).

### 2.3. Determination of Procyanidins by the Phloroglucinolysis Method

The extraction and measurement of procyanidins by the phloroglucinol test were performed according to the method by Lachowicz et al. [7]. Phloroglucinolysis was analyzed using a liquid chromatograph (Waters, Milford, MA, USA) consisting of a diode array, scanning fluorescence detectors, and a column manager. Separation was carried out using a Cadenza CD C18 column (75 mm × 4.6 mm, 3 μm) kept at 15 °C. For phloroglucinolysis investigation, the following solvent system (mobile phase A (25 mL of acetic acid and 975 mL of water) and mobile phase B (acetonitrile)) was applied. Fluorescence was recorded at emission wavelength 360 nm and excitation wavelength 278 nm. The calibration curves and quantification were evaluated using standards: (−)-epicatechin, (+)-catechin, (−)-epicatechin-phloroglucinol, and (+)-catechins-phloroglucinol. The degree of polymerization was analyzed by evaluating the molar ratio of all the flavan-3-ol units. All measurements were noted three times and expressed as mg/100 g d.w.

### 2.4. Determination of Isoprenoids (Carotenoids, Tocopherols, and Chlorophylls)

The extraction and analysis of isoprenoids (carotenoids, chlorophylls, and tocopherols) were performed according to the method by Stinco et al. [12]. Isoprenoid analyses were performed by RRLC on an Agilent 1260 system (Agilent, Waldbronn, Germany) equipped with a UV–VIS diode array detector, which was set at 285 nm for phytoene and tocopherols, 350 nm for phytofluene, 410 nm for ζ-carotene and pheophytin A, 430 nm for chlorophyll A and pheophytin B, 472 nm for lycopene, and 450 nm for the rest of the CARS (α-carotene, β-carotene, β-cryptoxanthin, capsanthin, lutein, violaxanthin, and zeaxanthin) and chlorophyll b. Separation was accomplished on a C30 column (150 mm × 4.6 mm I.D. 3 μm particle size; YMC Europe, Dinslaken, Germany) kept at 28 °C with a guard precolumn (10 mm × 4.0 mm I.D. 3 μm particle size; YMC Europe, Dinslaken, Germany). The analysis was performed in triplicate and expressed as mg/100 g d.w.

### 2.5. Determination of Organic Acids

The organic acid content was tested by the HPLC-RI method [11]. The chromatographic equipment SYKAM (Eresing, Germany) consisting of sample injector S5250, pump system S1125, column oven S4120, and RI detector S3590 was used. Separation was carried out using a Polymer IEX H column (6 µm, 250 mm × 8 mm; SETREX). Separation was achieved with a mobile phase of 1.5 mM sulfuric acid in water in isocratic mode. The flow rate was 0.5 mL/min at a column temperature set at 90 °C. The volume of the injected sample was 20 µL, and 30 min was needed to complete the analysis. The analysis was performed in triplicate and expressed as g/100 g d.w.

### 2.6. Analysis of Antioxidant Activity

Antiradical activity (ABTS) and reducing power (FRAP) tests were performed, as previously described by Re et al. [13] and Benzie and Strain [14]. Briefly, 10 µL of the supernatant was mixed with 990 µL of ABTS or FRAP. After 10 min of reaction, absorbance was measured at 734 nm for ABTS and 593 nm for FRAP. Determinations by ABTS and FRAP methods were performed using a UV-2401 PC spectrophotometer (Shimadzu, Kyoto, Japan) [5]. All antioxidant assays were performed in triplicate and expressed as mmol of TE per 100 g d.w.

### 2.7. Inhibitory of Biological Activity

Antihyperglycemic activity, α-glucosidase and α-amylase inhibitory activity, and anti-obesity pancreatic lipase inhibitory activity of the sample were determined, as described previously by Podsędek et al. [15] and Nickavar et al. [16]. The IC_50_ of the material was obtained from 1 mL of the reaction substance relative to the percentage inhibition. All results were presented as the average of three replicates.

### 2.8. Statistical Analysis

Statistical analysis, two-way ANOVA, Tuckey’s test, and PCA were performed using Statistica version 13.3 (StatSoft, Kraków, Poland), and significant differences (*p* < 0.05) were determined between mean parameters values.

## 3. Results and Discussion

### 3.1. Evaluation of Organic Acids

**Figure 1:**
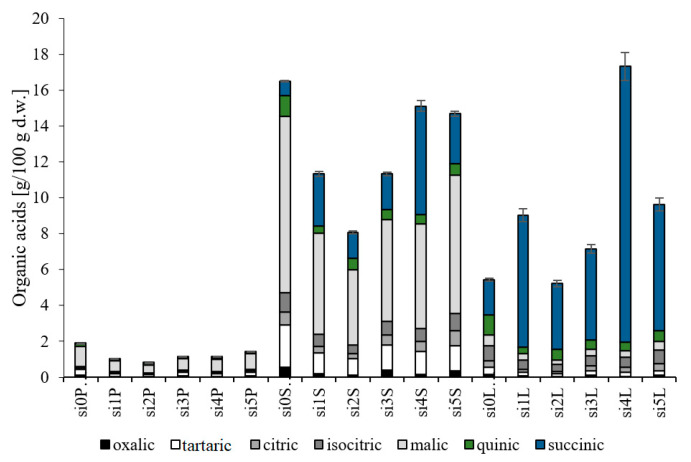
Organic acid profile and amount in fruit skin + pulp, seeds, and leaves of cherry silverberry biotypes. Explanation: si0P-si5P, fruit skin + pulp; si0S-si5S, seeds; and si0L-si5L, leaves.

The analysis of organic acids allows the determination of sensory attributes and health benefits of raw materials and food products. We investigated the contents of oxalic, tartaric, citric, isocitric, malic, quinic, and succinic acids in the fruit skin + pulp, seeds, and leaves of six biotypes of cherry silverberry (Figure 1). The analysis of organic acids indicated that succinic acid predominated in the leaves, whereas malic acid predominated in the fruit skin + pulp and seeds (with their contents accounting for 57%, 50%, and 75% of total organic acids, respectively), which is consistent with the results reported by other authors for cherry silverberry wine [17]. Both malic and succinic acids are natural metabolites involved in the Krebs cycle. They are used as acidity regulators and are responsible for the enhancement of the anti-oxidative potential of the raw materials they occur in. The average total organic acid content ranged from 1.24 to 12.83 g/100 g d.w. and was 10.0 and 7.0 times lower in the fruit skin + pulp than in the seeds and leaves, respectively, of the six cherry silverberry biotypes tested. Similar tendencies were found by Kolniak-Ostek [18] in pear fruit components. In addition, the contents of these organic acids in cherry silverberry wine were 17.0, 11.0, and 1.6 times lower than in the seeds, leaves, and fruit skin + pulp of *E. multiflora* Thunb., respectively [17]. The average amount of acids in the seeds of cherry silverberry was similar to that in red, white, and black currant and gooseberry and was 1.3, 1.6, 2.0, 2.7, and 5.6 times higher than in chokeberry, elderberry, bilberry, blackberry, and goji berry, respectively [19]. In addition, this amount in fruit skin + pulp was similar to that in goji berry and black mulberry [19]. Strong correlations were confirmed between the contents of organic acids and the anti-oxidative activity at *r* = 0.756 in ABTS assay and *r* = 0.748 in FRAP assay, as well as between antidiabetic activity and α-glucosidase-inhibiting activity (*r* = 0.657). In the case of compounds with antioxidative potential, organic acids showed a lower correlation, reaching *r* = 0.415. The results enable concluding that seeds, i.e., waste material being a component of pomace left after processing, represent the best source of organic acids among all studied components of cherry silverberry. This indicates that cherry silverberry by-products can be a good source of health-promoting compounds and an excellent additive to newly designed functional food.

### 3.2. Identification of Polyphenolic Compounds

Identification of polyphenols in six new cherry silverberry biotypes was performed with the LC-MS-TQD method (Table 1) in multiple reaction monitoring (MRM) mode on specific MS transitions. Identification allowed for tentative recognition of up to 63 compounds and 2 unidentified compounds as quercetin and kaempferol derivatives: 38 polyphenolic compounds were identified in the leaves; 14 in the seeds (3 less because they were repeated), and 11 in fruit skin + pulp. Owing to such an effective technique, all compounds were identified for the first time in the tested biotypes, and 17 polyphenolic compounds were identified for the first time in the seeds and leaves of cherry silverberry. Tentative identification was carried out in negative ion mode, whereas qualitative analysis of compounds was based on the reference standard, MS data, fragmentation patterns (MS/MS), and literature data.

#### 3.2.1. Phenolic Acids

Peaks 1 and 2 were identified in the leaves as quinic acid (*m/z* 191) and 3-*p*-coumaroylquinic acid (*m/z* 337), respectively, based on the standard and were previously reported in the fruits of pear [20], cherry elaegnus [7], and red grapes by Kapusta et al. [11]. Compound 14 was identified in the leaves and fruit skin + pulp as sinapic acid*-O*-hexoside, presented a neutral loss of 162 Da (hexose moiety), and was based on the main ion (*m/z* = 385) and MS/MS (*m/z* = 223). This compound was identified for the first time.

#### 3.2.2. Flavonols

In the present study, 52 flavonol derivatives were characterized as quercetin, isorhamnetin, and kaempferol flavonol derivatives. Among them, 12 compounds were quercetin derivatives (with a precursor ion at *m/z* = 301), 3 compounds were isorhamnetin derivatives (with the main fragment at *m/z* = 315), and 28 compounds were kaempferol derivatives (with the main fragment at *m/z* = 285). UV spectra of analyzed peaks demonstrated absorptions typical of these derivatives, where the maximum absorption at band I was 315–359 nm and at band II was 207–280 nm [21]. In turn, determination of sugar moieties was performed by classifying them as pentoside, hexoside, and/or deoxyhexoside, which corresponded to the losses of −132, −162, and/or −308 units from the molecular ions [22,23]. Among the identified compounds, there were also acetylated compounds (42 units) and combinations with caffeic acid (136 Da) and *p*-coumaric acid (176 Da) [11].

In addition, 36 compounds showed the presence of precursors of kaempferol aglycone [7,24,25,26,27], and 7 compounds were detected for the first time in the fruits of cherry silverberry [24,25,26,27]. Moreover, 16 polyphenolic compounds showed the presence of precursors of quercetin and isorhamnetin aglycone, and 5 compounds were detected for the first time in the fruits of cherry silverberry [7]. Among the identified flavonols, 11 compounds and 2 unspecified compounds were recorded for the first time in *E. multiflora* Thunb., especially in the seeds and leaves. Based on mass fragmentation, UV spectra, and standards, as well as literature data concerning saffron [28], compound 41 was identified as kaempferol di-hexoside. In addition, compounds such as kaempferol 3-*O*-(6′′-*p*-coumaryl)-galactoside and kaempferol 3-*O*-(6′′-*p*-coumaryl)-glucoside were previously identified in *Tiliae flos* by Toker et al. [29] but for the first time in the seeds. Compound 5 detected in the seeds with the MS/MS fragment on the pseudomolecular ion present at *m/z* = 609, 447, and 285 was identified as kaempferol-tri-hexoside (*m/z* = 771) [28]. Compounds 57 and 59 lost 42 Da, pointing to their acetyl moiety, as implied by Kolniak-Ostek [18]. These compounds formed the [M-H]- ion at *m/z* = 581 and 547 with a single MS/MS ion at *m/z* = 285. These derivatives were tentatively identified in the leaves as eriodictyol glycoside-pentoside and kaempferol malonyl-glucuronide. Compounds 47 and 60 had the main ion at *m/z* = 603 and *m/z* = 891 and only a single base peak at *m/z* = 285 and *m/z* = 301. The second fragmentation path corresponded to the loss of three hexose residues. Compounds such as quercetin 3-*O*-rutinoside (*m/z* = 609), kaempferol rhamnoside-rutinoside (*m/z* = 739), and quercetin-rhamnoside-glucopyranoside-rhamnoside (*m/z* = 769) detected in the leaves were previously found in sea buckthorn [30], *Tilia Americana* [31], and *Elaeagnus rhamnoides* L. [32]. The signals are indicative of kaempferol derivatives (present in the leaves) and quercetin derivatives (present in the seeds). However, the exact identification of these compounds is impossible without thorough fragmentation.

#### 3.2.3. Hydrolysable Tannins

Chromatographic analysis showed the presence of eight hydrolysable tannin derivatives determined just in the seeds. Compounds such as trigalloyl-hexoside were identified based on the main ion at *m/z* = 635 and a fragmentation ion at *m/z* = 421 and 169 and *m/z* = 465 and 331. The compound that had the [M-H]^–^ ion at *m/z =* 951 and the MS/MS fragment ion at *m/z =* 907 and 783 was identified as trigalloyl-hexahydroxydiphenoyl. The compound digalloyl-gallagyl-hexoside was identified based on the major ion at *m/z* = 1085 and the MS/MS ion at *m/z =* 765, 633, and 451. Compounds whose Rt was 4.22 and 4.37 min had the [M-H]^–^ ion at *m/z =* 787 and the fragment ion at *m/z =* 635, 617, and 301 and were identified as tetragalloyl-hexoside. The final compounds belonging to hydrolysable tannins were identified as pentagalloyl-hexoside with *m/z =* 939 and the fragment ion at *m/z =* 787, 635 and 301. These hydrolysable tannin derivatives have been previously noted in the literature [33], but they were determined here in cherry silverberry, especially in the seeds, for the first time.

### 3.3. Quantification of Polyphenols

**Figure 2:**
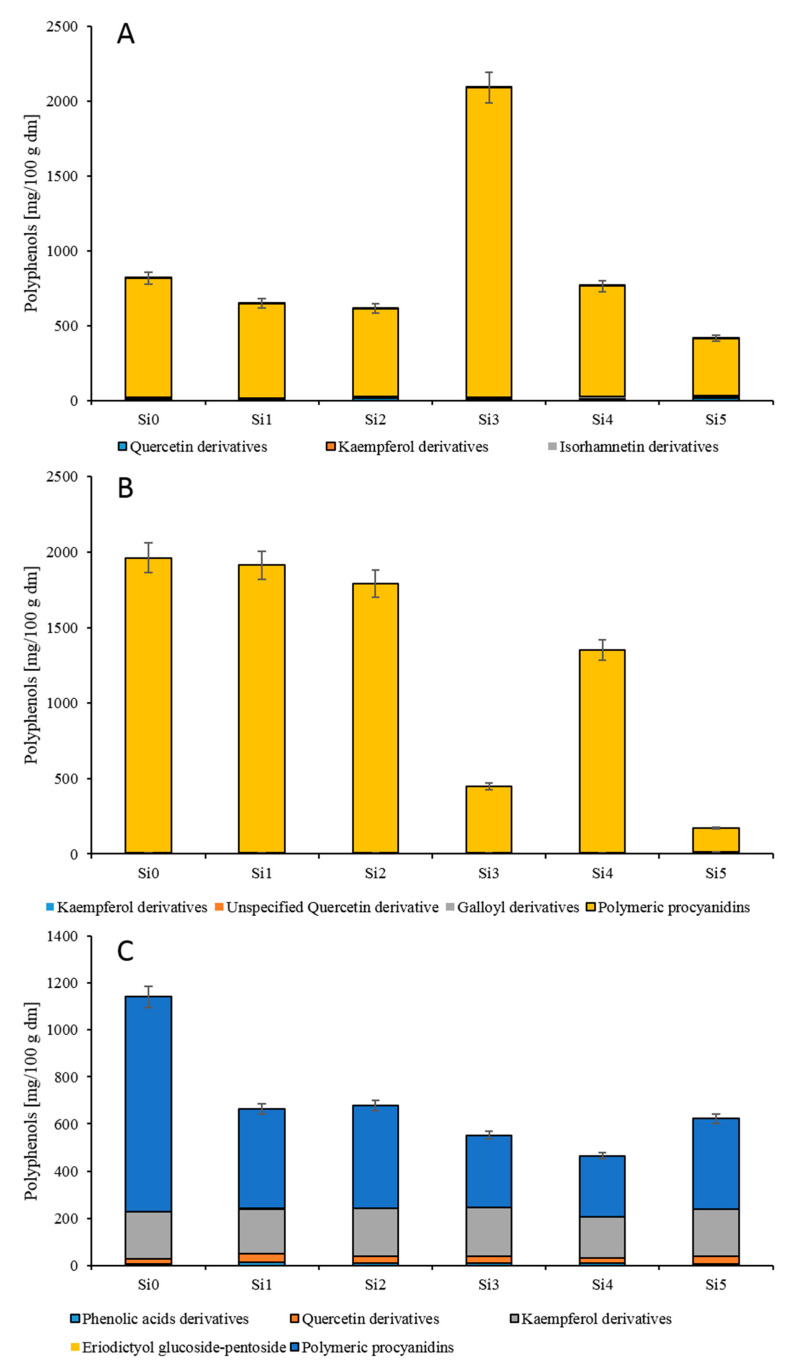
Polyphenolic compound content in the fruit pulp + skin (**A**), seeds (**B**), and leaves (**C**) of cherry silverberry (mg/100 g d.w.).

As presented in Figure 2 and Supplementary Material Tables S1–S3, the greatest variety of polyphenols was noted in the leaves and seeds compared with the fruit skin + pulp. The content of polyphenols in the tested parts of cherry silverberry ranged from 171.57 (si5) to 1951.01 (si0) mg/100 g d.w. in the seeds, from 464.21 (si4) to 1142.03 (si0) mg/100 g d.w. in the leaves, and from 417.02 (si5) to 819.04 (si0) mg/100 g d.w. in the fruit skin + pulp. According to Yoon et al. [34], the content of polyphenols determined in the leaves of *E.s multiflora* Thunb. was 805.60 mg/100 d.w. and was similar to that assayed in our study. Further, the values of polyphenols in Jahidka and Sweet Scarlet cultivars of *E. multiflora* Thunb. were 904.65 and 1268.90 mg/100 g d.w., and the average content was similar and 1.5 times higher than that in the fruit skin + pulp of tested biotypes, respectively [7]. The total polyphenolic compound content determined in sea buckthorn fruits (*Hippophae rhamnoides* L.) and gummi (*Elaeagnus indica*) was, on average, 4.0 and 7.0 times lower, respectively, than in the fruit skin + pulp of cherry silverberry (*E. multiflora* Thunb.) [35,36]. Studies on polyphenolic compounds in pear parts proved that their content in the seeds is 978.50 mg/100 g d.w., in the fruit skin + pulp is 234.2 mg/100 g d.w., and in the leaves is 5326.70 mg/100 g d.w. These values are 1.3 and 2.5 times lower and 7.7 times higher, respectively, compared to the tested parts of the cherry silverberry [18]. However, the content of polyphenolic compounds tested in the fruit skin + pulp and leaves of cherry silverberry was 6.0 and 17.0 times lower than in the fruit and leaves of cranberry, respectively [37].

The main group of polyphenols in the tested fruit skin + pulp, leaves, and seeds of cherry silverberry was polymeric procyanidins [37]. This group accounted for over 66.0–95.0% of total polyphenols identified in cherry silverberry. The content of polymeric procyanidins in the analyzed seeds ranged from 160 (si5) to 1951 (si0) mg/100 g d.w. and was, on average, 2.8 times higher than in the leaves and fruit skin + pulp. The content of procyanidins in cherry silverberry was similar to that determined in pear leaves [38] and gummi fruits and leaves [7] and also significantly lower than in saskatoon berry and cranberry fruits [37,39]. The average degree of polymerization in the fruit skin + pulp was 12.0, which was 1.2 and 2.5 times lower than in the seeds and leaves, respectively, and also similar to the degree of polymerization determined in grape and gummi fruits [40]. However, procyanidins are the most desirable components of nutraceuticals due to their higher antioxidant activity compared to flavonols and phenolic acids and due to their positive impact on human health [7].

The second group of polyphenolic compounds identified in cherry silverberry was flavonols, accounting for around 3.5–33.0% of total polyphenols identified in cherry silverberry. In the examined fruit skin + pulp, the presence of quercetin, kaempferol, and isorhamnetin derivatives was identified, but in the leaves and seeds, only quercetin and kaempferol derivatives were detected. Predominant compounds in the leaves and seeds were kaempferol derivatives and in the fruit skin + pulp quercetin derivatives. The average content of flavonols was 224.86 mg/100 g d.w. in the leaves, 23.64 mg/100 g d.w. in the fruit skin + pulp, and 6.09 mg/100 g d.w. in the seeds. In gummi fruit and leaves also [7], the concentration of flavonols was higher in the leaves (5.3 times) than in the fruits, and the average content of these compounds was 1.2 and 2 times higher compared to the leaves and fruit skin + pulp of new biotypes, respectively. In pear fractions [18], the highest content of flavonols was recorded in the leaves, followed by the seeds and fruit skin + pulp, while 4.0 times more flavonols were recorded in the cherry silverberry fruit skin + pulp than in the seeds. In addition, the fruit skin + pulp of *E. multiflora* Thunb. contained 3.3 times more flavonols than the fruits of *Elaeagnus indica* [35]. Flavonoid compounds are important because they show health-promoting and anti-oxidative properties [18,35].

The third group of polyphenolics was phenolic acids found in the leaves and seeds, accounting for around 2% of total polyphenols identified in cherry silverberry (three compounds). These phenolic acids were representatives of hydroxycinnamic acids [41]. Their content ranged from 4.92 (si0) to 12.34 (si1) mg/100 g d.w. in the leaves and from 0.57 (si1) to 2.39 (si4) mg/100 g d.w. in the fruit skin + pulp. The predominant phenolic acid was 3-*p*-coumaroyloqunic acid, followed by synapoyl acid*-O-*hexoside, which accounted for around 44% and 41% of total acids, respectively. Their average content was 3.78 and 3.51 mg/100 g d.w., respectively. The content of hydroxycinnamic acids in cherry silverberry was similar to that in different pear fruit parts [18] and gummi fruits [7], as well as being significantly lower than in cranberry fruits [37]. The presence of hydroxycinnamic acids is important because they are responsible for the protection of the low-density lipoprotein (LDL) fraction from oxidative changes and thereby inhibit atherogenesis and show anti-oxidative properties [18,41].

Hydrolysable tannins were the smallest group of polyphenols quantified in the seeds of cherry silverberry and accounted for around 1.5% of all polyphenols detected in the seeds. Their average content was 3.48 mg/100 g d.w. Hydrolysable tannins are compounds that induce apoptosis and reduce cell division (anticancer activity), and mainly occur in tea [33].

The obtained results show that the distribution and concentration of phenolic compounds in the six biotypes of cherry silverberry are closely related to the tested fractions. On the changelity of phenolic acid, flavonols, polymeric procyanidin, and hydrolysable tannins in plant tissues (in anatomical and morphological fractions of plants) influenced by different factors such as sunlight, weather conditions, temperature, and UV light [18].

### 3.4. Evaluation of Carotenoids, Chlorophylls, and Tocopherols

As presented in Table 2, Table 3 and Table 4, up to 18 isoprenoids were detected in the samples. Nine compounds were identified in the leaves, twelve in the seeds, and ten in the fruit skin + pulp. The distribution of carotenoids, chlorophylls, and tocopherols in the seeds and fruit skin + pulp has not yet been described. Chlorophylls were only detected in the leaves; tocopherol was detected in the seeds and the fruit skin + pulp. Among all identified carotenoids, only β-carotene was present in all tested fractions. Zeaxanthin and α-carotene were found only in the leaves; lutein and (9Z)-β-carotene were detected in the leaves and seeds. Phytoene and several geometrical isomers of lycopene were detected only in the seeds and the fruit skin + pulp of all new biotypes. The lycopene content in the fruit skin + pulp was ~23-fold higher than in seeds. Among the identified lycopene derivatives, (all-E)-lycopene was predominant and accounted for ~90% of all lycopene isomers. The results were in line with those reported in Sweet Scarlet and Jahidka cultivars of cherry silverberry [7]. Pheophytin b and zeaxanthin were detected for the first time in the leaves of cherry silverberry. However, α-tocopherol and (9Z)-β-carotene were detected for the first time in the seeds and fruit skin + pulp. Lachowicz et al. [7] identified eight carotenoids in the fruits of Sweet Scarlet and Jahidka cultivars of cherry silverberry. In the current study, lutein was detected in the seeds but not in the fruit skin + pulp. The best sources of lutein were the leaves, its content being ~143-fold higher, on average, than in the seeds, which agrees well with a previous study on other cultivars [7]. The content of lutein in the leaves (30.71–42.61 mg/100 g d.w.) and seeds (0.20–0.30 mg/100 g d.w.) of the six new biotypes was similar. The average content of α-tocopherol was 2.5 times higher in the fruit skin + pulp compared with the seeds. In addition, the content of phytoene was higher in the fruit skin + pulp and was five times higher compared with the seeds; β-carotene was predominant in the seeds and was 3.5 times higher than in the fruit skin + pulp. In the leaves, the predominant compounds among chlorophylls were chlorophylls a and b, accounting for 69% to 52% (for si5 to si2) and for 44% to 29% (for si2 to si5), respectively, of all chlorophylls. Our study showed a possible relationship between the type of chlorophylls and biotypes; thus, the more the amount of chlorophyll a, the less the amount of chlorophyll b, and vice versa. This relationship was not noticed in the study by Lachowicz et al. [7] in the leaves of cherry silverberry of the Sweet Scarlet and Jahidka cultivars.

The contents of chlorophylls and carotenoids were significantly dependent on the tested fractions and between biotypes (Table 2, Table 3 and Table 4). Similar observations were made between the anatomical parts of saskatoon berry [39]. The content of chlorophylls in the leaves was between 300 for si2 and 482 mg/100 g d.w. for si5, whereas the content of carotenoids detected in the leaves was from 56.75 for si0 to 96.28 mg/100 g d.w. for si1. The levels of total chlorophylls were three times more than the levels of carotenoids. Our results for foliar chlorophylls were, on average, four times lower compared with Sweet Scarlet and Jahidka and three times lower for carotenoids [7]. In addition, the amount of carotenoids in the fruit skin + pulp was between 95.69 and 170 mg/100 g d.w. and in the seeds was from 12.55 to 15.26 mg/100 g d.w. for si4 to si5. These levels were, on average, two times higher than in the fruit skin + pulp of saskatoon berry and similar to the seeds [39]. In addition, the average amount of carotenoids was nine times higher in the fruit skin + pulp than in the seeds. According to [42], the level of carotenoids in faveleira seeds oil was 0.03 mg/100 g and in chia seeds oil was 0.54 mg/100 g [42]. Thus, the seeds of cherry silverberry appear as an interesting carotenoid-containing by-product for industrial use. The average amount of carotenoids detected in the fruit skin + pulp was around 3 and 1.4 times higher than in the fruits of Sweet Scarlet and Jahidka cultivars, respectively, of cherry silverberry, whereas the content was 2 and 6 times lower, respectively, in the seeds [7].

In summary, some noticeable differences in the levels of carotenoids, tocopherols, and chlorophylls have been found between different parts and biotypes. The major carotenoids found are among the main dietary carotenoids for humans [43], which is interesting in the context of health promotion through diet and for the development of diverse products intended for human consumption.

### 3.5. Evaluation of Antioxidant Capacity

This study also included FRAP and ABTS assays that were conducted for the fruit skin + pulp, seeds, and leaves of six biotypes of *E. multiflora* Thunb. (Supplementary Material Table S4). The results obtained indicate significant differences in the values of the antioxidant capacity in the fruit skin + pulp, seeds, and leaves of selected new biotypes. The antioxidative capacity of fruit skin + pulp in cherry silverberry was, on average, 3.97 mmol TE/100 g d.w. according to the FRAP test and 7.25 mmol TE/100 g d.w. according to ABTS assay. These values were around 3.4 and 3.3 times (FRAP assay) and 3.8 and 3.6 times (ABTS assay) lower compared to these noted in the seeds and leaves, respectively, similar to the study by Kolniak-Ostek [18] on pear composition. The FRAP value was 1.2 times lower than the antioxidant capacity in all fruits of cherry silverberry tested by Bieniek et al. [1]. The average antioxidant capacity of the analyzed biotypes of cherry silverberry ranged between 58.92 (si0) and 8.34 mmol TE/100 g d.w. (si4) when tested with the FRAP method, as well as from 29.16 (si0) to 16.35 mmol TE/100 g d.w. (si4) when analyzed with the ABTS test. Moreover, the antioxidant capacity of saskatoon berry seeds investigated by Lachowicz [39] was in line with that determined for the seeds of cherry silverberry. In addition, the antioxidant capacity of cranberry leaves examined by Oszmiański et al. [37] was, on average, 45.17 mmol TE/100 g d.w., which is in line with the results reported for leaves in our work. Furthermore, the indicated capability for free radical reduction is probably due to the positive correlation between antioxidative compounds, which is consistent with the findings of other authors [41]. According to [44], the average antiradical activity and reducing power of the seeds of the grape cvs. Sangye (*Vitis ficifolia* Bunge.), white grape (*V. xunyangensis* P. C. H), and mao grape (*V. quinquangularis* Rehd.) was similar and of the seeds of black pearl grape and purple grape was 2.8 and 1.4 times and 3.8 and 1.8 times lower, respectively, than of the seeds of cherry silverberry determined in our study. However, the antiradical activity and reducing power determined in the skin of purple and white grapes was 2.3 and 3.5 times and 3.0 and 1.5 times higher, respectively, compared with the fruit skin + pulp of *E. multiflora* Thunb. [44]. However, as reported by Pantelić et al. [45], the average antiradical activity of the skin of white and red grape cvs. Pinot Gris and Cabernet Franc was comparable, red grape cvs. Sangiovese and Prokupac was 1.5 times higher, and white grape cvs. Riesling and Sauvignon Blanc was around 2 times lower than that in the fruit skin + pulp of *E. multiflora* Thunb. [45].

### 3.6. Evaluation of Inhibitory Biological Activity

Type II diabetes causes impairment of insulin synthesis by the pancreas, thereby leading to increased glycemia. The absorption of simple sugars should be controlled by inhibitors of enzymes responsible for sugar hydrolysis in the gastrointestinal tract. In turn, obesity and absorption of lipids should be controlled by pancreatic lipase inhibitors [46,47]. Therefore, the antidiabetic activity of *E. multiflora* Thunb. fruit parts was measured as the inhibitory activity against α-amylase, α-glucosidase, and pancreatic lipase. The tested parts of the new biotypes of cherry silverberry presented significant differences regarding their antidiabetic activity (Supplementary Material Table S1). The enzymatic inhibition against α-amylase and α-glucosidase in the fruit skin + pulp was, on average, 24.6 and 32.3 IC_50_ (mg/mL), respectively, whereas that against pancreatic lipase was, on average, 74.9 IC_50_ (mg/mL), and it was 3.0 times more effective than the antidiabetic activity of the seeds and leaves. The highest inhibition of the tested enzymes was noted for the fruit skin + pulp of the Si5 biotype (17.0 and 23.7 mg/mL against α-amylase and α-glucosidase, respectively), while the highest obesity inhibition was confirmed for the fruit skin + pulp of the Si4 biotype (69.0 mg/mL against pancreatic lipase). The antidiabetic activity of the tested *E. multiflora* fruit skin + pulp was similar to that noted for the extract of *Elaeagnus umbellata* [46]. According to Saltan et al. [47], the inhibitory activity of *Elaeagnus angustifolia* leaf extracts toward α-glucosidase and α-amylase was similar to that of the leaf extract of *E. umbellata*. In addition, this inhibitory activity measured in the fruit skin + pulp and seeds was similar to that in the seeds and skin of the grape Pinot Noir cultivar [48]. Bhardwaj et al. [49] reported α-glucosidase inhibitory activity of 14.5% to 43.09 % for seabuckthorn leaf extracts, whereas the leaf extracts of *Hippophae rhamnoides* L. (belonging to the family *Eleagnaceae*), extracted by *n*-butanol, showed an α-glucosidase inhibitory effect of 82% [50]. You et al. [51] noted that the α-glucosidase and pancreatic lipase inhibitory activities of the seeds of muscadine were 1.91 and 34.41 IC_50_ (mg/mL), respectively. Moreover, in the red Norton (*Vitis aestivalis*) grape skin, the α-glucosidase inhibitory activity was noted at IC_50_ = 10.5 μg/mL and was 32-fold more effective than acarbose in inhibiting yeast α-glucosidase [52]. The results obtained in comparison with the available data show a high potential for the use of the tested parts of cherry silverberry as by-products in various branches of the food and/or cosmetics industries. The obtained results were correlated with the phenol content (α-amylase *r =* 0.732, α-glucosidase *r =* 0.683, pancreatic lipase *r =* 0.360). The strong correlation with polyphenolic compounds is induced by the antidiabetic effect of these compounds occurring due to their binding with digestive enzymes [15]. Thus, the best antidiabetic properties were exhibited by the fruit skin + pulp, whose components migrate to juice during the pressing process. In addition, cherry silverberry juice can be used to produce a powdered functional additive. Furthermore, sugars can be removed to enhance its antidiabetic effect.

### 3.7. Principal Component Analysis (PCA)

**Figure 3:**
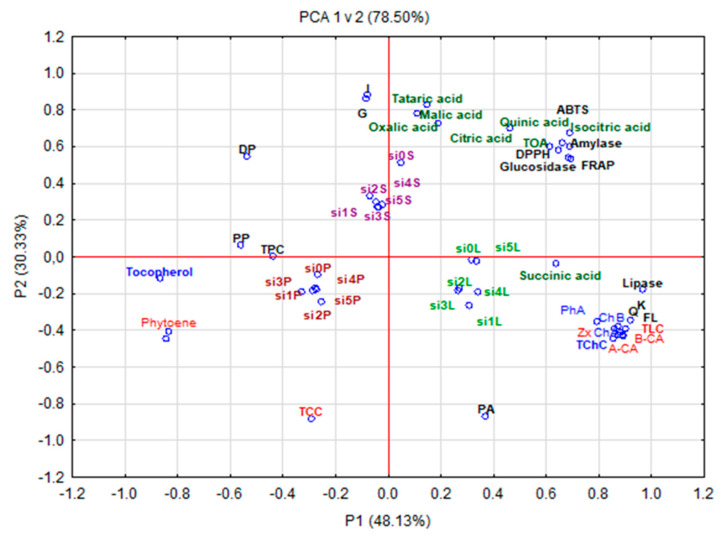
PCA map showing the relationship among chemical composition and biological activities of 6 new biotypes in the peel, seeds, and leaves of cherry silverberry. Explanation: si0P-si5P, the fruit skin + pulp; si0S-si5S, the seeds; si0L-si5L, the leaves; I, isorhamnetin; G, hydrolysable tannin; K, kaempferol; Q, quercetin; FL, sum of flavonoids; PP, polymeric procyanidins; DP, degree of polymerization; TPC, sum of polyphenols; PA; sum of phenolic acids; TCC; sum of carotenoids; TLC, sum of lycopene; A-CA, α-carotene; B-CA, β-carotene; Zx, zeaxanthin; ChB, chlorophyll b; ChA, chlorophyll a; TChC; sum of chlorophylls.

PCA showed correlations between the organic acid content, polyphenolic compound content, and antioxidant activity of the fruit parts of *E. multiflora* Thunb. (Figure 3). The PCA graph presented 73.90% of the whole variation, where PC1 and PC2 explained 44.16% and 29.76% of intergroup variances, respectively. This statistical analysis presented three major groups. Group I comprised leaves opulent in phenolic acids; the sum of flavonols (especially kaempferol and quercetin); the sum of lycopene; carotenoids such as β-carotene, α-carotene, and zeaxanthin; the sum of chlorophylls (especially chlorophyll b, chlorophyll a, and pheophytin a), and succinic acid and exhibiting antidiabetic activity against lipase. Group II included seeds rich in the sum of organic acids and their components, hydrolysable tannins, quercetin, and isorhamnetin and showing a high degree of polymerization as well as high antioxidant capacity and antidiabetic activity confirmed by α-amylase and α-glucosidase inhibition. Group III consisted of fruit peel with a high sum of polyphenolic compounds, the sum of tocopherols, polymeric procyanidins, and phytoene, as well as the sum of carotenoids. The arrangement of variables for all groups points to a positive correlation between the analyzed parameters.

## 4. Conclusions

In summary, owing to such an effective technique as liquid chromatography, 88 compounds (including 7 organic acids, 1 tocopherol, 13 carotenoids, 4 chlorophylls, 52 flavonols, 8 hydrolysable tannins, and 3 phenolic acids) were recorded and 17 polyphenolic compounds and 3 isoprenoids were identified for the first time in the seeds and leaves of *E. multiflora* Thunb. In total, 55 compounds were identified in the leaves, 36 in the seeds, and 31 in the fruit skin + pulp. The predominant group of polyphenols was polymeric procyanidins (66–95% of total polyphenolics), whereas the predominant isoprenoids were chlorophyll b and (all-E)-lycopene. The leaves contained significant levels of phenolic acids, the sum of flavonols (kaempferol and quercetin), lycopene, β-carotene, α-carotene, and chlorophylls, as well as highly inhibited lipase activity. The seeds were rich in organic acids, hydrolysable tannins, and isorhamnetin and showed a high degree of polymerization, high antioxidant capacity, and high inhibition of α-amylase and α-glucosidase. In addition, the fruit skin + pulp had high contents of total polyphenolic compounds, carotenoids, and phytoene, as well as polymeric procyanidins. In vitro antidiabetic activity measured as α-glucosidase, α-amylase, and lipase inhibitory activity of cherry silverberry fractions was positively correlated with the contents of polyphenolic compounds, antioxidant activity, and isoprenoids. Given the results of studies on diverse bioactive compounds, fruit fractions can be used to develop products intended for human consumption, such as functional foods, nutritional supplements, and nutricosmetics, among others.

## Figures and Tables

**Table 1:** Tentative identification of polyphenolic compounds in the seeds, fruit skin + pulp, and leaves of cherry silverberry.

Peak No.	Tentative Identification	Rt (min)	[M-H]^−^ (*m/z*)	[M-H]^−^ MS/MS (*m/z*)	UV–VIS (nm)	Leaves	Seeds	Fruit Skin + Pulp	Literature
1	Quinic acid	1.05	191	172	262	X			[7,11,20]
2	3-*p*-Coumaroyloqunic acid	1.33	337	191	276	X			[7,11,20]
3	Methyl-quercetin 3-*O*-rhamnoside-pentoside	1.94	609	463/331/299	317	X			[7]
4	Quercetin glycoside-pentoside-glycoside	2.53	757	595/463/301	255/352	X			[7]
5	Kaempferol-tri-hexoside	2.57	771	609/447/285	267/350		X		[28]
6	Kaempferol 3-*O*-rutinoside-7-*O*-glucoside	2.58	755	609/447/285	267/350	X			[7,26,27]
7	Kaempferol-tri-hexoside-rhamnoside	2.65	917	771/609/285	269/350		X		[7]
8	Kaempferol di-rhamnoside-di-glucoside	2.78	901	755/609/447/285	266/350	X			[7]
9	Quercetin pentoside-rutinoside	2.8	741	609/463/301	255/350	X			[7]
10	Kaempferol 7-*O*-pentoside	2.87	417	285	281/340	X			[7,24,25]
11	Kaempferol 3-O-rhamnoside	3.14	431	285	267/325	X			[7,24,25]
12	Kaempferol glucoside-rutinoside	3.17	755	431/285	266/319	X			[7,26,27]
13	Kaempferol glucopyranoside-rhamnoside-deoxyhexose	3.30	915	593/285	267/350		X		[7]
14	Sinapic acid-*O*-hexoside	3.36	385	223	325	X		X	
15	Kaempferol rhamnoside-dihexoside	3.37	917	771/285	274/322		X		[7]
16	Quercetin rhamnoside-pentoside-rutinoside	3.39	887	609/579/301	255/352			X	[7]
17	Kaempferol pentoside-rhamnoside-rutinoside	3.43	887	755/609/447/285	264/338	X			[7]
18	Kaempferol glucoside-di-rhamnoside	3.43	755	593/431/285	266/347		X		[7]
19	Quercetin 3-*O*-rutinoside	3.44	609	463/301	255/352	X			[30,31,32]
20	Quercetin rhamnoside-pentoside-rhamnoside	5.45	887	741/595/433/301	255/355	X			[7]
21	Quercetin 3-*O*-rhamnoside	3.49	447	301	255/326	X			[7,26,27]
22	Kaempferol rhamnoside-rutinoside	3.53	739	593/447/285	265/326	X			[30,31,32]
23	Trigalloyl-hexoside	3.53	635	421/169	274		X		[33]
24	Kaempferol rhamnoside-pentoside-rutinoside	3.66	871	725/563/431/285	266/340	X			[7]
25	Kaempferol pentoside-rutinoside	3.67	725	579/417/285	267/350			X	[7]
26	Trigalloyl-hexoside	3.68	635	465/313	276		X		[33]
27	Kaempferol pentoside-rutinoside	3.71	725	579/417/285	265/345	X			[7]
28	Kaempferol hexoside-pentoside-rhamnose	3.76	725	563/431/285	267/350		X		[7]
29	Kaempferol rhamnoside-pentoside	3.79	563	417/285	265/328	X			[7]
30	Kaempferol 3-*O*-rutinoside	3.84	593	447/285	265/334	X			[7,26,27]
31	Kaempferol glucopyranoside-dihexoside	3.86	785	609/447/285	267/350		X		[7]
32	Quercetin pentoside-rutinoside	3.91	741	609/433	255/350			X	[7]
33	Kaempferol di-rhamnoside-di-glycoside	3.97	901	755/609/447/285	264/339	X			[7]
34	Trigalloyl-hexahydroxydiphenoyl	4.02	951	907/783	274		X		[33]
35	Quercetin 3-*O*-rhamnoside-7-*O*-pentoside	4.12	595	433/301	255/350			X	[7]
36	Digalloyl-gallagyl-hexoside	4.14	1085	765/633/451	272		X		[33]
37	Quercetin-*O*-glucoside-*O*-pentoside	4.16	595	463/301	255/345	X			[7]
38	Kaempferol di-rhamnoside-glucoside	4.19	739	593/447/285	264/345	X			[7,26,27]
39	Tetragalloyl-hexoside	4.22	787	635/617/301	272		X		[33]
40	Kaempferol rhamnoside-rutinoside	4.24	739	593/285	265/340			X	[30,31,32]
41	Kaempferol-di-hexoside	4.31	609	447/285	267/350		X		[28]
42	Quercetin-tri-rhamnoside	4.33	739	593/447/301	255/355			X	[7]
43	Tetragalloyl-hexoside	4.37	787	635/617/301	277		X		[33]
44	Kaempferol di-rhamnoside-glucoside	4.37	739	593/447/285	265/338	X			[7,26,27]
45	Quercetin di-rhamnose	4.47	593	447/301	255/347	X			[7]
46	Quercetin-rhamnoside-glucopyranoside-rhamnoside	4.48	769	593/447/301	255/352			X	[30,31,32]
47	Unspecified quercetin derivative	4.48	603	301	252/366		X		
48	Pentagalloyl-hexoside	4.57	939	787/635/301	269		X		[33]
49	Kaempferol pentoside-rhamnoside-glucuronide	4.59	739	563/417/285	265/324	X			[7]
50	Kaempferol di-rhamnoside-hexoside	4.66	739	593/447/285	265/319	X			[7]
51	Kaempferol pentoside-di-rhamnoside	4.80	709	577/431/285	265/339	X			[7]
52	Kaempferol di-rhamnose	4.89	577	431/285	264/341	X			[7]
53	Pentagalloyl-hexoside	4.89	939	787/635/301	274		X		[33]
54	Kaempferol-3-*O*-glucoside	5.01	447	285	264/319	X			[7,24,25]
55	Isorhamnetin-7-*O*-rutinoside	5.09	623	477/315	253/358			X	[7]
56	Kaempferol glucoside-glucuronide	5.12	623	447/285	264/317	X			[7]
57	Eriodictyol glucoside-pentoside	5.19	581	285	265/315	X			[18]
58	Isorhamnetin-3-*O*-glucoside	5.20	477	315	256/380			X	[7]
59	Kaempferol malonyl-glucuronide	5.27	547	285	265/315	X			[18]
60	Unknown derivative of Kaempferol	5.63	891	285	269/325	X			
61	Isorhamnetin 3-*O*-(6”malonyl)-glucuronide-rhamnoside	5.71	723	491/315	270/350			X	[7]
62	Kaempferol 3-*O*-rhamnoside	5.94	431	285	264/325	X			[7,24,25]
63	Kaempferol 3-*O*-(6”-*p*-coumaryl)-galactoside	6.87	593	447/285	267/312	X		X	[29]
64	Kaempferol 3-*O*-(6”-caffeoyl)-glucoside	7.00	623	447/285	264/321	X		X	[7]
65	Kaempferol 3-*O*-(6”-*p*-coumaryl)-glucoside	7.11	593	447/285	267/315	X		X	[29]

^X^ It is mean that the identified compounds are available in the tested fraction.

**Table 2:** Quantification of carotenoids and chlorophylls (mg/100 g d.w.) in the leaves of cherry silverberry.

No.	Compounds	Si0	Si1	Si2	Si3	Si4	Si5
Chlorophylls							
1	Chlorophyll b	127 ± 3a ^1,2^	161 ± 8b	132 ± 1a	139 ± 6a	164 ± 1b	139 ± 7a
2	Chlorophyll b-d	17.18 ± 1.27a	15.36 ± 1.03a	8.38 ± 0.79b	8.58 ± 0.97b	8.36 ± 0.35b	7.67 ± 0.43b
3	Chlorophyll a	184 ± 7a	297 ± 17bc	156 ± 9a	173 ± 12a	263 ± 10b	333 ± 22c
4	Pheophytin b	4.21 ± 0.31a	1.70 ± 0.04bc	2.82 ± 0.18d	3.11 ± 0.19d	2.07 ± 0.17c	1.24 ± 0.16b
Sum		334 ± 8D	477 ± 20B	300 ± 11F	325 ± 10E	439 ± 9C	482 ± 23A
Carotenoids							
5	Lutein	30.71 ± 1.31a	42.10 ± 0.42b	35.40 ± 0.93c	34.58 ± 1.06c	42.61 ± 0.53b	39.20 ± 1.19d
6	Zeaxanthin	3.43 ± 0.32a	2.11 ± 0.08b	2.24 ± 0.09b	2.20 ± 0.20b	2.78 ± 0.12c	2.18 ± 0.21b
7	α-Carotene	10.19 ± 0.29a	27.73 ± 0.28b	18.37 ± 0.72c	18.76 ± 1.06c	23.97 ± 0.22d	21.95 ± 0.92e
8	β-Carotene	10.58 ± 0.58a	20.89 ± 0.26b	16.08 ± 0.56c	19.90 ± 0.54b	21.09 ± 0.22b	19.61 ± 0.97b
9	9Z β-carotene	1.83 ± 0.05a	3.46 ± 0.17b	2.66 ± 0.15c	4.16 ± 0.25d	3.46 ± 0.04b	3.30 ± 0.13b
	Sum (mg/100 g d.w.)	56.75 ± 2.52F	96.28 ± 0.79A	74.75 ± 1.75E	79.60 ± 2.54D	93.90 ± 0.98B	86.23 ± 2.64C

^1^ Values are means ± standard deviation; ^2^ a–f: Means-SD followed by different letters within the same line represent significant differences (*p* < 0.05).

**Table 3:** Quantification of tocopherols and carotenoids (mg/100 g d.w.) in the seeds of cherry silverberry.

No.	Compounds	Si0	Si1	Si2	Si3	Si4	Si5
Tocopherol							
1	α-Tocopherol	2.48 ± 0.35ab ^1,2^	2.97 ± 0.33ab	2.95 ± 0.22ab	3.32 ± 0.25b	2.00 ± 0.21a	2.43 ± 0.60ab
Carotenoids							
2	Phytoene	0.06 ± 0.01a	0.32 ± 0.06b	0.05 ± 0.02a	0.08 ± 0.01a	0.07 ± 0.01a	0.16 ± 0.01c
3	Lutein	0.20 ± 0.03a	0.30 ± 0.02a	0.29 ± 0.02a	0.24 ± 0.04a	0.29 ± 0.14a	0.23 ± 0.01a
4	β-Carotene	0.39 ± 0.04a	0.83 ± 0.05b	0.78 ± 0.07b	0.70 ± 0.10bc	0.42 ± 0.03a	0.63 ± 0.01c
5	9Z β-carotene	0.05 ± 0.01a	0.10 ± 0.01b	0.11 ± 0.02b	0.11 ± 0.01b	0.06 ± 0.01ac	0.09 ± 0.01bc
6	di-*Z* lycopene	0.04 ± 0.01a	0.06 ± 0.02a	0.06 ± 0.01a	0.05 ± 0.01a	0.04 ± 0.01a	0.06 ± 0.01a
7	(15*Z*)-lycopene	0.07 ± 0.01a	0.08 ± 0.01ab	0.09 ± 0.01ab	0.09 ± 0.01ab	0.08 ± 0.01ab	0.10 ± 0.01b
8	(13*Z*)-lycopene	0.26 ± 0.02a	0.46 ± 0.03b	0.36 ± 0.01cd	0.33 ± 0.04ce	0.29 ± 0.02ae	0.40 ± 0.02bd
9	di-*Z* lycopene	0.17 ± 0.01a	0.26 ± 0.04b	0.21 ± 0.03ab	0.22 ± 0.03ab	0.23 ± 0.03ab	0.20 ± 0.01ab
10	(9*Z*)-lycopene	0.15 ± 0.03a	0.27 ± 0.09b	0.18 ± 0.02ab	0.12 ± 0.03a	0.11 ± 0.01a	0.15 ± 0.01a
11	(all-*E*)-lycopene	5.15 ± 0.63a	3.74 ± 0.41b	3.73 ± 0.07b	5.32 ± 0.31a	4.03 ± 0.25b	5.22 ± 0.21a
12	(5*Z*)-lycopene	0.81 ± 0.06a	1.89 ± 0.34b	0.99 ± 0.14a	0.94 ± 0.08a	1.07 ± 0.13a	0.95 ± 0.11a
13	∑ Lycopene isomers	6.66 ± 0.74ab	6.77 ± 0.26ab	5.63 ± 0.15c	7.08 ± 0.32b	5.85 ± 0.06ac	7.08 ± 0.30b
	Sum (mg/100 g d.w.)	14.02 ± 1.56	15.08 ± 0.47	12.48 ± 0.30	15.30 ± 0.69	12.55 ± 0.13	15.26 ± 0.63

^1^ Values are means ± standard deviation; ^2^ a–f: Means-SD followed by different letters within the same line represent significant differences (*p* < 0.05).

**Table 4:** Quantification of tocopherols and carotenoids (mg/100 g d.w.) in the fruit skin + pulp of cherry silverberry.

No.	Compounds	Si0	Si1	Si2	Si3	Si4	Si5
Tocopherol							
1	α-Tocopherol	9.79 ± 0.49a ^1,2^	9.93 ± 0.47a	3.70 ± 0.20b	7.07 ± 0.33c	6.76 ± 0.54c	3.31 ± 0.43b
Carotenoids							
2	Phytoene	0.81 ± 0.04a	1.04 ± 0.06b	1.08 ± 0.04b	1.20 ± 0.05c	0.44 ± 0.02d	0.86 ± 0.04a
3	β-Carotene	0.31 ± 0.02a	0.29 ± 0.02ab	0.30 ± 0.02	0.25 ± 0.01bc	0.21 ± 0.01c	0.30 ± 0.02a
4	di-*Z* lycopene	0.33 ± 0.02a	0.15 ± 0.01b	0.21 ± 0.01c	0.11 ± 0.01d	0.19 ± 0.01c	0.19 ± 0.01c
5	(15*Z*)-lycopene	0.60 ± 0.02a	0.66 ± 0.03a	0.47 ± 0.03b	0.70 ± 0.07a	0.60 ± 0.04a	1.01 ± 0.06c
6	(13*Z*)-lycopene	9.09 ± 0.20a	9.92 ± 0.39b	6.15 ± 0.17c	7.92 ± 0.15d	6.35 ± 0.22c	10.53 ± 0.42b
7	di-*Z* lycopene	0.30 ± 0.02a	0.39 ± 0.04a	0.60 ± 0.03b	0.68 ± 0.05b	0.80 ± 0.05c	1.68 ± 0.04d
8	(9*Z*)-lycopene	1.09 ± 0.09a	1.02 ± 0.08ab	0.72 ± 0.06cd	0.68 ± 0.05c	0.87 ± 0.06bd	1.17 ± 0.05a
9	(all-*E*)-lycopene	110 ± 1a	93.89 ± 1.83b	108 ± 1a	94.49 ± 0.96b	80.31 ± 1.28c	144 ± 1d
10	(5*Z*)-lycopene	4.89 ± 0.27a	13.75 ± 0.36b	6.15 ± 0.11a	6.10 ± 0.39a	5.93 ± 0.42a	10.21 ± 0.87d
11	∑ Lycopene isomers	126 ± 1a	120 ± 2b	123 ± 1ab	111 ± 1c	95.05 ± 1.65d	169 ± 2e
	Sum (mg/100 g d.w.)	127 ± 2B	121 ± 2D	124 ± 1C	112 ± 1E	95.69 ± 1.66F	170 ± 3A

^1^ Values are means ± standard deviation; ^2^ a–f: Means-SD followed by different letters within the same line represent significant differences (*p* < 0.05).

## Data Availability

The data presented in this study are available on request from the corresponding author.

## Supplementary Materials

The following are available online at https://www.mdpi.com/article/10.3390/antiox10060849/s1, Table S1. Polyphenolic compound content in seeds of cherry silverberry (mg/100 g d.w.); Table S2. Polyphenolic compound content in fruit skin + pulp of cherry silverberry (mg/100 g d.w.); Table S3. Polyphenolic compound content in leaves of cherry silverberry (mg/100 g d.w.); Table S4. Antioxidant activity and in vitro enzymatic activity of new biotypes of cherry silverberry.
