# Supplementary material for: Distribution of Polyphenolic and Isoprenoid Compounds and Biological Activity Differences between in the Fruit Skin + Pulp, Seeds, and Leaves of New Biotypes of Elaeagnus multiflora Thunb
Source: Antioxidants (Basel). 2021 May 26;10(6):849. doi: 10.3390/antiox10060849 (PMC8229477; doi:10.3390/antiox10060849)
Supplement: Supplementary file 1 [file antioxidants-10-00849-s001.zip › antioxidants-1193283-supplementary.pdf]

Article

Distribution of Polyphenolic and Isoprenoid Compounds and Biological Activity Differences between in the Fruit Skin + Pulp, Seeds, and Leaves of New Biotypes of *Elaeagnus multiflora* Thunb.

Sabina Lachowicz-Wiśniewska [1]*, Ireneusz Kapusta [2], Carla M. Stinco [3], Antonio J. Meléndez-Martínez [3], Anna Bieniek [4], Ireneusz Ochmian [5] and Zygmunt Gil [1]

1 Department of Fermentation and Cereals Technology, Wrocław University of Environmental and Life Science, Chełmońskiego 37, 51-630 Wroclaw, Poland; sabina.lachowicz@upwr.edu.pl
2 Department of Food Technology and Human Nutrition, Faculty of Biology and Agriculture, Rzeszow University, Zelwerowicza 4, 35-601 Rzeszow, Poland; ikapustai@ur.edu.pl
3 Food Colour and Quality Laboratory, Area of Nutrition and Food Science, Universidad de Sevilla, 41012 Seville, Spain; cstinco@us.es; ajmelendez@us.es
4 Department of Horticulture, University of Warmia and Mazury, Prawocheńskiego 21, 10-720 Olsztyn, Poland; anna.bieniek@uwm.edu.pl
5 Department of Horticulture, West Pomeranian University of Technology in Szczecin, Słowackiego 17, 71-434 Szczecin, Poland; ireneusz.ochmian@zut.edu.pl
* Correspondence: sabina.lacowicz@upwr.edu.pl

Table S1. Polyphenolic compound content in the seeds of cherry silverberry (mg/100 g d.w.).

Compounds	Si0	Si1	Si2	Si3	Si4	Si5
Kaempferol-tri-hexoside	0.21 ± 0.04b	0.16 ± 0.03b	Tr	Tr	0.43 ± 0.09a	Tr
Kaempferol-tri-hexoside-rhamnoside	0.06 ± 0.01b	0.05 ± 0.01b	Tr	Tr	0.17 ± 0.03a	Tr
Kaempferol glucopyranoside-rhamnoside-deoxyhexose	0.12 ± 0.02b	0.09 ± 0.02b	Tr	Tr	0.40 ± 0.08a	Tr
Kaempferol-rhamnoside-dihexoside	Tr	Tr	Tr	Tr	0.31 ± 0.06a	Tr
Kaempferol-glucoside-dirhamnoside	3.45 ± 0.69c	2.62 ± 0.52e	3.98 ± 0.80b	3.97 ± 0.79b	3.06 ± 0.61d	4.55 ± 0.91a
Trigalloyl-hexoside	1.51 ± 0.30b	1.15 ± 0.23c	1.47 ± 0.29b	1.29 ± 0.26c	0.97 ± 0.19d	1.81 ± 0.36a
Trigalloyl-hexoside	0.55 ± 0.11c	0.42 ± 0.08d	0.67 ± 0.13b	0.45 ± 0.09d	0.80 ± 0.16a	0.59 ± 0.12c
Kaempferol-hexoside-pentoside-rhamnose	1.20 ± 0.24b	0.91 ± 0.18c	1.15 ± 0.23b	1.05 ± 0.21c	0.42 ± 0.08d	1.31 ± 0.26a
Kaempferol glucopyranoside-dihexoside	0.67 ± 0.13a	0.51 ± 0.10b	0.64 ± 0.13a	0.49 ± 0.1c	0.14 ± 0.03d	0.68 ± 0.14a
Trigalloyl-hexahydroxydiphenoyl	0.10 ± 0.02a	0.08 ± 0.02b	0.13 ± 0.03a	0.08 ± 0.02b	0.07 ± 0.01b	0.06 ± 0.01c
Digalloyl-gallagyl-hexoside	0.13 ± 0.03c	0.10 ± 0.02c	0.23 ± 0.05b	0.09 ± 0.02d	0.37 ± 0.07a	0.14 ± 0.03c
Tetragalloyl-hexoside	0.12 ± 0.02b	0.09 ± 0.02c	0.13 ± 0.03b	0.11 ± 0.02b	0.04 ± 0.01d	0.16 ± 0.03a
Kaempferol-di-hexoside	0.44 ± 0.09b	0.33 ± 0.07c	0.40 ± 0.08b	0.31 ± 0.06c	0.48 ± 0.10a	0.19 ± 0.04d
Tetragalloyl-hexoside	0.13 ± 0.03c	0.10 ± 0.02c	0.08 ± 0.02d	0.32 ± 0.06a	0.16 ± 0.03b	0.09 ± 0.02d
Unspecified quercetin derivative	0.37 ± 0.07a	0.28 ± 0.06b	0.28 ± 0.06b	0.30 ± 0.06b	0.13 ± 0.03d	0.25 ± 0.05c
Pentagalloyl-hexoside	0.36 ± 0.07a	0.27 ± 0.05c	0.38 ± 0.08a	0.36 ± 0.07a	0.04 ± 0.01d	0.34 ± 0.07b
Pentagalloyl-hexoside	0.79 ± 0.16c	0.60 ± 0.12d	0.84 ± 0.17b	0.77 ± 0.15c	0.47 ± 0.09e	0.89 ± 0.18a
Polymeric procyanidins	1951 ± 3a	1904 ± 3b	1781 ± 3c	437 ± 1e	1342 ± 2d	160 ± 1f
Degree of polimeryzation	13.96 ± 2.79a	10.61 ± 2.12d	11.24 ± 2.25c	12.46 ± 2.49b	6.78 ± 1.36e	2.04 ± 0.41f
Sum (mg/100 g d.w.)	1961 ± 3a	1912 ± 38b	1791 ± 3c	447 ± 1e	1351 ± 2d	171 ± 1f

[1] Values are means ± standard deviation; [2] a–f: Means-SD followed by different letters within the same line represent significant differences ($p < 0.05$).

Table S2. Polyphenolic compound content in the skin + pulp of cherry silverberry (mg/100 g d.w.).

Compounds	Si0	Si1	Si2	Si3	Si4	Si5
Quercetin-rhamnoside-pentoside-rutinoside	2.13 ± 0.43e	1.70 ± 0.34f	3.82 ± 0.76b	2.57 ± 0.51c	2.31 ± 0.46d	4.8 ± 0.96a
Sinapic acid-*O*-glucoside	0.71 ± 0.14e	0.57 ± 0.11f	2.20 ± 0.44b	1.21 ± 0.24d	2.39 ± 0.48a	1.89 ± 0.38c
Kaempferol-pentoside-rutinoside	0.35 ± 0.07d	0.28 ± 0.06e	0.50 ± 0.10b	0.41 ± 0.08c	0.33 ± 0.07d	0.57 ± 0.11a
Quercetin-pentoside-rutinoside	1.33 ± 0.27e	1.06 ± 0.21f	2.44 ± 0.49c	1.80 ± 0.36d	2.63 ± 0.53b	3.03 ± 0.61a

Quercetin-3-*O*-rhamnoside-7-*O*-pentoside	1.18 ± 0.24d	0.94 ± 0.19e	1.31 ± 0.26c	0.82 ± 0.16f	3.11 ± 0.62a	2.29 ± 0.46b
Kaempferol-rhamnoside-rutinoside	1.85 ± 0.37d	1.48 ± 0.30e	2.99 ± 0.60b	2.35 ± 0.47c	1.32 ± 0.26f	3.28 ± 0.66a
Quercetin-tri-rhamnoside	0.82 ± 0.16d	0.66 ± 0.13f	1.49 ± 0.30a	1.28 ± 0.26b	0.74 ± 0.15e	1.11 ± 0.22c
Quercetin-rhamnoside-glucopyranoside-rhamnoside	4.56 ± 0.91d	3.64 ± 0.73f	6.18 ± 1.24b	5.00 ± 1.00c	4.29 ± 0.86e	7.24 ± 1.45a
Isorhamnetin-7-*O*-rutinoside	1.18 ± 0.24d	0.94 ± 0.19f	1.33 ± 0.27c	1.11 ± 0.22e	2.84 ± 0.57a	1.63 ± 0.33b
Isorhamnetin-3-*O*-glucoside	1.04 ± 0.21d	0.83 ± 0.17e	1.77 ± 0.35a	1.26 ± 0.25c	1.42 ± 0.28b	1.48 ± 0.3b
Isorhamnetin 3-*O*-(6″malonyl)-glucuronide-rhamnoside	1.41 ± 0.28d	1.13 ± 0.23e	2.39 ± 0.48b	1.78 ± 0.36c	3.46 ± 0.69a	2.37 ± 0.47b
Kaempferol-3-*O*-(6″-p-coumaryl)-glucoside	1.87 ± 0.37c	1.49 ± 0.30d	2.28 ± 0.46a	1.95 ± 0.39b	1.29 ± 0.26e	1.86 ± 0.37c
Kaempferol 3-*O*-(6″-caffeoyl)-glucoside	0.59 ± 0.12c	0.47 ± 0.09d	0.71 ± 0.14b	0.56 ± 0.11c	1.54 ± 0.31a	0.57 ± 0.11c
Kaempferol-3-*O*-(6″-p-coumaryl)-glucoside	0.78 ± 0.16d	0.62 ± 0.12e	1.03 ± 0.21b	0.85 ± 0.17c	1.11 ± 0.22a	0.87 ± 0.17c
Polymeric procyanidins	799 ± 1b	632 ± 1d	586 ± 1e	2069 ± 4a	739 ± 1c	384 ± 1f
Degree of polimeryzation	12.62 ± 0.25b	10.08 ± 0.20d	9.46 ± 0.19e	11.23 ± 0.22c	10.65 ± 0.21d	16.43 ± 0.33a
Sum (mg/100 g d.w.)	819 ± 1b	648 ± 1d	617 ± 1e	2092 ± 4a	768 ± 1c	417 ± 1f

[1] Values are means ± standard deviation; [2] a–f: Means-SD followed by different letters within the same line represent significant differences ($p < 0.05$).

Table S3. Polyphenolic compound content in the leaves of cherry silverberry (mg/100 g d.w.).

Compounds	Si0	Si1	Si2	Si3	Si4	Si5
Qunic acid	1.03 ± 0.21d	1.76 ± 0.35a	1.35 ± 0.27b	1.18 ± 0.24c	0.97 ± 0.19d	1.20 ± 0.24c
3-*p*-Coumaroyloqunic acid	2.39 ± 0.48e	7.24 ± 1.45a	4.33 ± 0.87b	3.19 ± 0.64d	2.09 ± 0.42f	3.45 ± 0.69c
Methyl-quercetin 3-*O*-rhamnoside-pentoside	1.63 ± 0.33a	1.59 ± 0.32b	1.63 ± 0.33a	1.27 ± 0.25c	0.57 ± 0.11d	1.29 ± 0.26c
Quercetin glycoside-pentoside-glycoside	1.79 ± 0.36f	7.23 ± 1.45a	4.01 ± 0.8d	4.86 ± 0.97c	2.14 ± 0.43e	5.29 ± 1.06b
Kaempferol 3-*O*-rutinoside-7-*O*-glucoside	0.35 ± 0.07c	0.96 ± 0.19a	0.95 ± 0.19a	0.94 ± 0.19a	0.35 ± 0.07c	0.89 ± 0.18b
Kaempferol di-rhamnoside-di-glucoside	0.75 ± 0.15d	1.24 ± 0.25b	1.31 ± 0.26a	1.30 ± 0.26a	0.45 ± 0.09e	1.15 ± 0.23c
Quercetin pentoside-rutinoside	0.21 ± 0.04e	0.88 ± 0.18a	0.40 ± 0.08d	0.55 ± 0.11c	0.46 ± 0.09d	0.67 ± 0.13b
Kaempferol 7-*O*-pentoside	0.46 ± 0.09d	0.74 ± 0.15b	0.73 ± 0.15b	0.80 ± 0.16a	0.33 ± 0.07e	0.63 ± 0.13c
Kaempferol 3-*O*-rhamnoside	0.42 ± 0.08d	0.70 ± 0.14a	0.52 ± 0.10c	0.57 ± 0.11b	0.45 ± 0.09d	0.55 ± 0.11b
Kaempferol glucoside-rutinoside	1.52 ± 0.30a	1.26 ± 0.25b	1.29 ± 0.26b	1.51 ± 0.30a	1.02 ± 0.20c	1.18 ± 0.24c
Sinapic acid-*O*-glucoside	1.50 ± 0.30f	3.34 ± 0.67c	3.28 ± 0.66d	3.67 ± 0.73b	6.33 ± 1.27a	3.10 ± 0.62e
Kaempferol pentoside-rhamnoside-rutinoside	2.56 ± 0.51a	2.25 ± 0.45b	2.01 ± 0.40c	2.24 ± 0.45b	2.08 ± 0.42c	2.26 ± 0.45b
Quercetin 3-*O*-rutinoside	1.82 ± 0.36e	5.82 ± 1.16a	4.28 ± 0.86c	4.91 ± 0.98b	4.05 ± 0.81d	5.02 ± 1.00b
Quercetin rhamnoside-pentoside-rhamnoside	2.09 ± 0.42c	2.18 ± 0.44b	1.00 ± 0.20f	1.38 ± 0.28e	3.21 ± 0.64a	1.82 ± 0.36d
Quercetin 3-*O*-rhamnoside	0.84 ± 0.00c	1.08 ± 0.00b	1.00 ± 0.00b	1.03 ± 0.00b	1.74 ± 0.00a	1.08 ± 0.00b
Kaempferol rhamnoside-rutinoside	0.66 ± 0.01d	1.92 ± 0.04b	2.09 ± 0.04a	1.94 ± 0.04b	1.80 ± 0.04c	1.92 ± 0.04b
Kaempferol rhamnoside-pentoside-rutinoside	1.87 ± 0.00d	2.87 ± 0.01b	2.86 ± 0.01b	2.73 ± 0.01c	3.01 ± 0.01a	1.50 ± 0.00e
Kaempferol pentoside-rutinoside	24.31 ± 0.05d	27.04 ± 0.05c	28.9 ± 0.06b	27.06 ± 0.05c	38.56 ± 0.08a	27.87 ± 0.06c
Kaempferol rhamnoside-pentoside	1.86 ± 0.37d	2.44 ± 0.49a	2.04 ± 0.41c	2.42 ± 0.48a	2.30 ± 0.46b	2.40 ± 0.48a
Kaempferol 3-*O*-rutinoside	2.08 ± 0.00d	5.84 ± 0.01a	5.15 ± 0.01c	5.13 ± 0.01c	1.22 ± 0.00e	5.44 ± 0.01c
Kaempferol di-rhamnoside-di-glycoside	1.81 ± 0.36d	0.49 ± 0.10f	4.42 ± 0.88c	4.52 ± 0.90b	1.31 ± 0.26e	5.53 ± 1.11a
Quercetin-*O*-glucoside-*O*-pentoside	1.66 ± 0.33d	2.81 ± 0.56c	3.06 ± 0.61b	1.10 ± 0.22f	3.78 ± 0.76a	1.29 ± 0.26e
Kaempferol di-rhamnoside-glucoside	109.31 ± 2.19a	93.21 ± 1.86d	100.83 ± 2.02b	100.7 ± 2.01b	74.04 ± 1.48	96.59 ± 1.93c
Kaempferol di-rhamnoside-glucoside	3.44 ± 0.69b	1.15 ± 0.23e	1.24 ± 0.25d	2.96 ± 0.59c	3.96 ± 0.79a	1.18 ± 0.24e
Quercetin di-rhamnose	13.40 ± 2.68c	15.75 ± 3.15a	13.12 ± 2.62c	14.93 ± 2.99b	6.05 ± 1.21d	15.17 ± 3.03a
Kaempferol pentoside-rhamnoside-glucuronide	2.01 ± 0.40e	3.90 ± 0.78a	3.09 ± 0.62c	2.26 ± 0.45d	1.99 ± 0.40f	3.69 ± 0.74b
Kaempferol di-rhamnoside-hexoside	1.02 ± 0.20c	1.24 ± 0.25b	0.90 ± 0.18e	0.96 ± 0.19d	4.02 ± 0.80a	1.07 ± 0.21c
Kaempferol pentoside-di-rhamnoside	2.91 ± 0.58b	1.17 ± 0.23c	1.07 ± 0.21d	1.07 ± 0.21d	3.39 ± 0.68a	1.17 ± 0.23c
Kaempferol di-rhamnose	38.75 ± 0.78a	34.26 ± 0.69d	37.14 ± 0.74b	38.69 ± 0.77a	27.68 ± 0.55e	36.85 ± 0.74c
Kaempferol-3-*O*-glucoside	1.20 ± 0.24c	1.35 ± 0.27b	1.34 ± 0.27b	1.31 ± 0.26b	2.02 ± 0.40a	1.19 ± 0.24c
Kaempferol glucoside-glucuronide	0.41 ± 0.08f	2.12 ± 0.42c	2.27 ± 0.45b	1.96 ± 0.39d	1.51 ± 0.30e	2.34 ± 0.47a
Eriodictyol glucoside-pentoside	0.50 ± 0.10d	1.94 ± 0.39b	1.58 ± 0.32c	0.11 ± 0.02e	0.50 ± 0.10d	2.07 ± 0.41a
Kaempferol malonyl-glucuronide	0.39 ± 0.08c	0.39 ± 0.08c	0.35 ± 0.07d	1.75 ± 0.35a	0.34 ± 0.07d	0.50 ± 0.10b
Unknown derivatives of Kaempferol	0.25 ± 0.05e	1.62 ± 0.32a	0.58 ± 0.12c	0.66 ± 0.13b	0.32 ± 0.06d	0.25 ± 0.05e
Kaempferol 3-*O*-rhamnoside	0.29 ± 0.06d	0.27 ± 0.05d	1.16 ± 0.23a	0.70 ± 0.14b	0.29 ± 0.06d	0.51 ± 0.10c
Kaempferol 3-*O*-(6″-*p*-coumaryl)-galactoside	0.91 ± 0.18b	0.49 ± 0.10c	0.25 ± 0.05e	1.34 ± 0.27a	0.31 ± 0.06d	0.21 ± 0.04f
Kaempferol 3-*O*-(6″-caffeoyl)-glucoside	0.55 ± 0.11a	0.20 ± 0.04d	0.38 ± 0.08b	0.25 ± 0.05d	0.37 ± 0.07b	0.30 ± 0.06c
Kaempferol 3-*O*-(6″-*p*-coumaryl)-glucoside	0.27 ± 0.05c	0.03 ± 0.01e	0.16 ± 0.03d	0.32 ± 0.06b	0.16 ± 0.03d	0.41 ± 0.08a
Polymeric procyanidins	912 ± 2a	422 ± 1c	436 ± 1b	308 ± 1e	259 ± 1f	383 ± 1d
Degree of polimeryzation	6.57 ± 0.13a	4.97 ± 0.10c	5.8 ± 0.12b	4.88 ± 0.10c	2.99 ± 0.06d	2.66 ± 0.05e

Sum (mg/100 g d.w.)	1142 ± 2a	662 ± 1c	678 ± 2b	552 ± 1d	464 ± 2e	622 ± 1c

[1] Values are means ± standard deviation; [2] a–f: Means-SD followed by different letters within the same line represent significant differences ($p < 0.05$).

Table S4. Antioxidant activity and in vitro enzymatic activity of new cultivars of cherry silverberry.

Part	Biotypes	Antioxidant Activity (mmoL TE/100 g d.w.)		Antidiabetic Activity IC_{50} (mg/mL)		
		FRAP	ABTS	α-Amylase	α-Glucosidase	Pancreatic Lipase
Skin + Pulp	Si0	5.38 ± 0.02a	6.65 ± 0.33a	31.08 ± 0.06e	44.92 ± 0.09e	77.82 ± 0.16e
	Si1	2.06 ± 0.00d	4.80 ± 0.10d	23.40 ± 0.05d	26.87 ± 0.05c	75.84 ± 0.15d
	Si2	2.72 ± 0.01c	6.47 ± 0.13c	33.25 ± 0.07f	43.99 ± 0.09d	79.57 ± 0.16f
	Si3	4.69 ± 0.01b	7.70 ± 0.15b	19.71 ± 0.04c	22.78 ± 0.05b	69.05 ± 0.14b
	Si4	1.63 ± 0.00e	4.30 ± 0.09e	17.04 ± 0.03b	23.04 ± 0.05b	72.33 ± 0.14c
	Si5	1.38 ± 0.00e	3.55 ± 0.07f	13.04 ± 0.03a	18.04 ± 0.05a	59.33 ± 0.14a
Seeds	Si0	13.99 ± 0.03c	37.88 ± 0.76a	81.99 ± 0.16e	102.45 ± 0.20e	123.41 ± 0.25e
	Si1	14.60 ± 0.03b	31.56 ± 0.63b	67.50 ± 0.14b	86.71 ± 0.17c	105.67 ± 0.21b
	Si2	14.71 ± 0.03b	30.24 ± 0.60c	78.93 ± 0.16d	92.33 ± 0.18d	103.42 ± 0.21a
	Si3	11.68 ± 0.02d	23.56 ± 0.47e	75.41 ± 0.15c	92.91 ± 0.19d	107.88 ± 0.22c
	Si4	8.26 ± 0.02e	15.62 ± 0.31f	66.20 ± 0.13a	80.77 ± 0.16a	101.24 ± 0.20a
	Si5	17.39 ± 0.03a	28.35 ± 0.57d	65.18 ± 0.13a	83.49 ± 0.17b	111.52 ± 0.22d
Leaves	Si0	13.51 ± 0.03b	32.95 ± 0.66a	87.51 ± 0.18d	125.53 ± 0.25e	219.12 ± 0.44d
	Si1	10.15 ± 0.02d	21.48 ± 0.43e	65.89 ± 0.13c	78.28 ± 0.16c	213.55 ± 0.43c
	Si2	12.70 ± 0.03c	25.28 ± 0.51c	93.63 ± 0.19e	119.31 ± 0.24d	224.05 ± 0.45e
	Si3	10.94 ± 0.02e	24.52 ± 0.49d	55.50 ± 0.11b	68.40 ± 0.14b	194.43 ± 0.39a
	Si4	15.15 ± 0.03a	29.14 ± 0.58b	47.98 ± 0.10a	66.79 ± 0.13a	203.67 ± 0.41b
	Si5	15.95 ± 0.03a	21.22 ± 0.42e	47.98 ± 0.10a	66.79 ± 0.13a	203.67 ± 0.41b
Skin + Pulp	Mean	3.97^{c}	7.25^{c}	23.59^{a}	30.77^{a}	74.49^{a}
Seeds		13.44^{a}	27.87^{a}	72.54^{c}	89.78^{c}	108.86^{b}
Leaves		13.07^{b}	25.76^{b}	66.42^{b}	87.51^{b}	209.75^{c}

[1] Values are means ± standard deviation; [2] a–f: Means-SD followed by different letters within the same line represent significant differences ($p < 0.05$).
